# Intelligent non-colorimetric indicators for the perishable supply chain by non-wovens with photo-programmed thermal response

**DOI:** 10.1038/s41467-020-19676-y

**Published:** 2020-11-25

**Authors:** Luigi Romano, Alberto Portone, Maria-Beatrice Coltelli, Francesco Patti, Rosalba Saija, Maria Antonia Iatì, Giuseppe Gallone, Andrea Lazzeri, Serena Danti, Onofrio M. Maragò, Andrea Camposeo, Dario Pisignano, Luana Persano

**Affiliations:** 1 NEST, Istituto Nanoscienze-CNR and Scuola Normale Superiore, Piazza San Silvestro 12, I-56127 Pisa, Italy; 2 Department of Civil and Industrial Engineering, University of Pisa, Largo L. Lazzarino 2, I-56122 Pisa, Italy; 3 Dipartimento di Scienze Matematiche e Informatiche, Scienze Fisiche e Scienze della Terra, Università di Messina, Viale F. Stagno d’Alcontres 31, I-98166 Messina, Italy; 4 CNR-IPCF, Istituto Processi Chimico-Fisici, Viale F. Stagno D’Alcontres, 37, I-98158 Messina, Italy; 5 Department of Civil and Environmental Engineering, Massachusetts Institute of Technology, 33 Massachusetts Ave, Cambridge, MA 02142 USA; 6 Dipartimento di Fisica, Università di Pisa, Largo B. Pontecorvo 3, I-56127 Pisa, Italy

**Keywords:** Polymers, Environmental, health and safety issues, Polymers

## Abstract

Spoiled perishable products, such as food and drugs exposed to inappropriate temperature, cause million illnesses every year. Risks range from intoxication due to pathogen-contaminated edibles, to suboptimal potency of temperature-sensitive vaccines. High-performance and low-cost indicators are needed, based on conformable materials whose properties change continuously and irreversibly depending on the experienced time-temperature profile. However, these systems can be limited by unclear reading, especially for colour-blind people, and are often difficult to be encoded with a tailored response to detect excess temperature over varying temporal profiles. Here we report on optically-programmed, non-colorimetric indicators based on nano-textured non-wovens encoded by their cross-linking degree. This combination allows a desired time-temperature response to be achieved, to address different perishable products. The devices operate by visual contrast with ambient light, which is explained by backscattering calculations for the complex fibrous material. Optical nanomaterials with photo-encoded thermal properties might establish new design rules for intelligent labels.

## Abstract (web summary)

High-performance and low-cost indicators are important in food and cosmetics industry but market uptake is low due to several challenges such as toxicity, cost and unclear reading. Here, the authors report on optically-programmed, non-colorimetric indicators based on nanotextured organic non-wovens, encoded by controlling their cross-linking degree.

## Introduction

Perishable goods, such as foods, cosmetics, and drugs, spoil over time spontaneously, and at an accelerated pace upon increasing temperature; as such, their appropriate handling and storage are critically important for public safety^1,2^. A crucial piece of information for citizens is given by the expiration date, or date of minimum durability of a product, defined as the interval of time within which the properties, quality, and specifications of a good under proper storage conditions remains effective and safe for human consumption. Also, the suitable storage temperature must be present on the package leaflet and on the item labels as required, for instance, by the European Union for medicinal products^3^. However, stability tests allowing such data to be determined are based on the use of controlled temperatures, which is generally different from the real life, during which undesired temperature variations can occur by an enormous variety of issues during transportation, distribution, and storage. The consequences cause several million illnesses every year, including intoxication from improperly stored food in which high temperature induced enhanced growth rates of microbial pathogens (e.g., verocytotoxigenic *Escherichia coli, Listeria*, *Salmonella,* and *Campylobacter*)^4^, scombroid poisoning from histamine-contaminated fish^5^, and diseases related to suboptimal potency of deteriorated vaccines^6^. While the control over the supply cold chain is strictly regulated for the manufacturers, wholesalers, distributors, and transporters at each level of good distribution, even inappropriate handling by consumers and domestic storage conditions must be taken into account as potential sources of risk.

Time–temperature indicators (TTIs)^7–9^ are devices fabricated, for instance, in the form of labels or stickers that can largely mitigate these risks for the final user. They should be small and flexible to comply with items of any size and shape^2^, and preferably rely on a solid-state technology to avoid any possible leakage of fluid reactants. Electronics embedding radio-frequency identification tags and wireless antennas, whose resonant response evolves while they are conformed to spoiling food surfaces^10^, are elegant data loggers capable of transmitting quality information, but intelligent indicators providing a clear and user-friendly visual information of the overall time–temperature history of an item have the potential to induce a much better perception in consumers^7^, thus greatly improving general safety without affecting electronic waste. To this aim, an indicator must be based on a material showing a continuous and irreversible change of a physico-chemical property depending on the time–temperature profile undergone by the product with which it is coupled. This effect has been reported for a variety of colorimetric systems, including organic semiconductors^9^, plasmonic metal nanocrystals^8,11^, nanocomposites of chitosan and gold nanoparticles^12^, photochromic inks with temperature-dependent discoloration^13^, enzymatic indicators^14^, and microencapsulated microorganisms^15^. However, most of the TTIs have barely superficially penetrated the market. This is due to a number of reasons, such as potential unclear reading^7^, concern for the eventual diffusion of toxic reactants from the device, and high cost of the technology behind, which might account for 50–100% of the whole packaging cost^16^ and lead to increased product price. Also, colorimetric indicators can be unsuitable for color-blind people. Furthermore, indicator materials are generally highly difficult or impossible to be programmed, i.e. to be encoded with a tailored response of their components in order to detect excess temperature over varying temporal profiles, ranging from a few minutes up to several days to address the need of many different classes of perishable products (food, drugs, vaccines, etc.). While efforts towards programming have been focused on finely controlling the colloidal^11^ or polymer^17^ chemistry of involved components, the possibility of embedding response instructions by optical control might open a much more practical perspective for the extensive use of smart TTIs.

Here we report on photo-programmed, non-colorimetric indicators based on nano-textured organic non-wovens. A two-level information is incorporated in the devices by controlling the degree of cross-linking in organic fibers by initial system encoding through calibrated photo-exposure doses (which allows a desired time–temperature profile response to be achieved) and embedding readable patterns that emerge along with temperature exposure in a continuous way (which enables the device to operate by visual contrast with ambient light, in a highly user-friendly way).

## Results

### Electrospinning and photo-programming of organic non-wovens

**Fig. 1:**
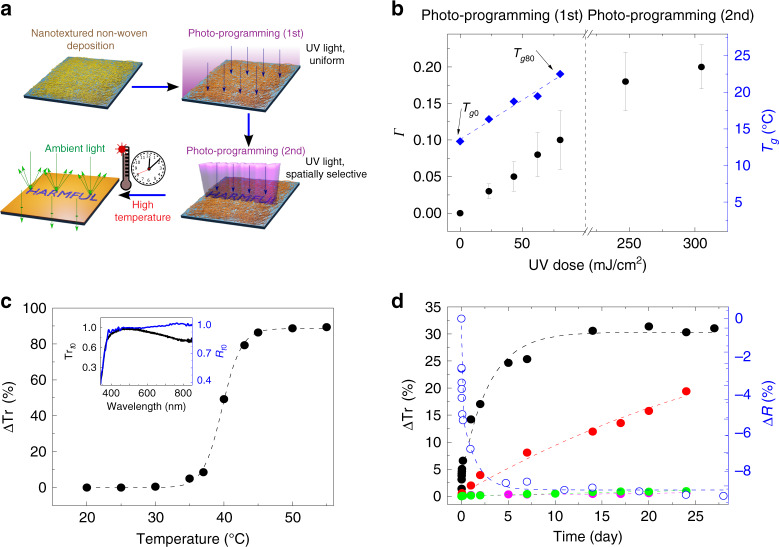
Photo-programming of thermal properties. **a** Schematic representation of the indicator photo-programming. SU-8 fibrous non-woven deposition and photo-programming (blue vertical rays) at calibrated optical dose and pattern activation. A warning sign will appear when the subsequently experienced time–temperature profile overcomes targeted programmed values (i.e., correct item storage conditions) by diffuse reflectance contrast (green rays). **b** Calculated average values of the cross-linking degree, Γ, at different exposure doses during photo-programming (left vertical scale). Data are averaged over at least three samples for each dose (error bars: SD). DSC inflection points (*T*_g_, right vertical scale) for the non-woven fabrics following the first step of photo-programming at different UV doses are also displayed. The blue line is a linear fit to the experimental data (diamonds). *T*_g0_ and *T*_g80_ refer to samples photo-programmed (step 1) with UV doses: 0 and 80 mJ/cm^2^, respectively. **c** Optical transmission change (dots, ΔTr) at *λ* = 510 nm, vs. temperature, for a fabric with first step of photo-programming at 80 mJ/cm^2^. The dashed line is a guide for the eye. Inset: Normalized Tr_*t*0_ (black line, left vertical scale) and *R*_*t*0_ (blue line, right vertical scale) spectra. Tr_*t*0_ and *R*_*t*0_ are the optical transmission and the diffuse reflectance measured at *t*_0_ = 0, respectively. *R*_*t*0_ was measured by means of an integrating sphere (see Methods). **d** Temporal variation of ΔTr (at *λ* = 510 nm, left vertical scale) for samples kept at *T*_amb_ (~ 20 °C, pink dots), 25 °C (green dots), 30 °C (red dots) and 35 °C (black dots). The corresponding optical reflectance change, Δ*R* (at 510 nm, empty dots, right scale), vs. time is also shown for samples at 35 °C. Dashed lines are guides for the eye.

The photo-programming steps of the indicators are illustrated in the scheme of Fig. 1a. Uniform and smooth fibers are electrospun from SU-8 3025, which is a negative epoxy resist largely used in optical and electron-beam lithography, by a proper choice of the spinning parameters (Figs. S1 and S2 in Supplementary Information). Compared to other organic compounds, SU-8 exhibits a high refractive index (∼1.59 in the visible spectral range), which has motivated its past use in waveguides incorporating quantum dots^18^. In the framework of the present work, this property is relevant to provide surfaces coated by SU-8 non-wovens with remarkable diffuse reflectance as better explained below. As-spun organic fibers are not stable over time, with melting observed within 30 min even at room temperature as shown in Figs. S3 and  S4. Therefore, in our process the non-woven is initially stabilized by a first photo-programming step, which is performed soon after spinning to promote SU-8 crosslinking by a calibrated UV exposure dose (<100 mJ/cm^2^) on all of the surface (top-right part of Fig. 1a). Then, a second photo-programming step is carried out that is spatially selective, i.e., a further UV exposure is performed on a part of the fabric through an optical mask (bottom-right part of Fig. 1a). The two consecutive illuminations are complementary in defining the properties and subsequent evolution of the programmed indicator, since the first one encodes the overall time–temperature behavior of the system through its thermal response, whereas the second provides further dose to design a pre-determined, non-colorimetric ultimate visual output.

In photocurable thermoset resins complete solidification induced by light-activated cationic polymerization normally occurs during post-exposure baking well above the glass transition temperature, *T*_g_ (∼50 °C in SU-8 uncured films). Very few reactions are expected to take place below the glass transition temperature due to the largely limited molecular motions^19^. Instead, at variance with the common lithographic processes, no post-exposure baking is performed here to promote resin crosslinking in the very thin organic fibers. The degree of cross-linking of the material might be affected by various mechanisms, including the fast interface dynamics impacting on the cooperative motion of monomers^20^, local chain restrictions^21^, and photothermal effects generated by the absorption of light^22,23^. With the purpose of investigating this behavior in depth, we analyze SU-8 non-woven fabrics before and after exposure by Fourier-transform infrared (FTIR) spectroscopy and differential scanning calorimetry (DSC), evidencing a correlation of the material properties with the dose shown in Fig. 1b. The FTIR spectra (Fig. S5) show peaks corresponding to the C–O stretching modes of the epoxy groups (at 862 and 911 cm^−1^), whose intensity decreases upon SU-8 cross-linking, and peaks at 1508 and 1607 cm^−1^, corresponding to C–C stretch modes of the aromatic ring, whose intensities do not vary during the polymerization process^24,25^. The absorption values of the peaks at 911 and 1607 cm^−1^ can be used to estimate the degree of polymerization upon UV exposure (Fig. 1b), through the expression, Γ = 1−[(*A*_*E*_/*A*_*R*_)/(*A*_*E*0_/*A*_*R*0_)], where *A*_*E*_ (*A*_*E*0_) and *A*_*R*_ (*A*_*R*0_) denote the intensities of the peak at 911 cm^−1^ and of the reference peak at 1607 cm^−1^, before (*A*_*E*0_, *A*_*R*0_) and after (*A*_*E*_, *A*_*R*_) exposure, respectively^26,27^. Γ is found to increase up to a maximum around 10% upon increasing the exposure dose in the first photo-programming step (0–80 mJ/cm^2^), highlighting that a fine control is achievable for the cross-linking degree of the non-woven material and, consequently, for the device time–temperature response. For the as-spun non-woven, the glass transition temperature (*T*_g0_ in Fig. 1b) is 13 °C (Fig. S6), i.e., below room temperature, which is in agreement with the low stability and melting behavior observed in unexposed fibers. The *T*_g_ value moves to higher temperatures (Fig. S6), increasing roughly linearly upon UV exposure at low dose, at a rate of about 0.1 °C×cm^2^/mJ (Fig. 1b). In addition, it is worth noticing that the UV doses used here in the first photo-programming step are well below those usually employed in lithographic processes on the resist compound (150–250 mJ/cm^2^)^28^, thus making the material still reactive to UV for the second step. Indeed, Γ is additionally doubled after the second photo-programming step (>200 mJ/cm^2^), highlighting remarkably increased cross-linking (Fig. 1b).

Due to the varied cross-linking degree, we find that each programmed non-woven is very sensitive to a specific working temperature, *T*_w_, in terms of its optical (light-scattering) properties. This aspect is exemplified in Fig. 1c, where we show how the optical transmittance, Tr, measured at an incident light wavelength (*λ*) of 510 nm for non-wovens exposed by 80 mJ/cm^2^ (*T*_w_ ≅ 38 °C), changes upon heating at different temperatures. Here, ΔTr is given by Tr  − Tr_*t*0_, where Tr is measured upon keeping the samples at each given temperature for 60 min, and Tr_*t*0_ is the transmittance at *t*_0_ = 0, i.e., immediately after photo-programming. Data show an almost stable optical transmittance up to 30 °C (ΔTr ~ 0.6 %) followed by a rapid increase of the system transparency for temperatures in the interval 30–45 °C (ΔTr ~ 80%) and a new plateau condition (ΔTr ~ 90%) above 45 °C. This behavior is related to the rapid increase in the mobility and the formation of a viscous flow, following chain disentanglements in the fibrous material as promoted by heating. An analogous mechanism has been recently exploited with self-healing thermoplastic polyurethanes^17^. The corresponding transmittance and diffuse (hemispherical) reflectance (*R*) spectra for the as-spun (*t* = 0) material are displayed in the inset of Fig. 1c, highlighting a wavelength-independent (white) behavior in the range 400–700 nm, which makes the system optimal for use with ambient light (Fig. S7). Long-term experiments are additionally performed by maintaining the system at constant temperature such as, 20, 25, 30, and 35 °C up to 25 days (Fig. 1d). The material is stable at *T* ≤ 25 °C, with no significant change found in the transmittance, thus setting an upper limit for prior-use storage temperature of this specific system. Instead, a remarkable response in the long term starts to be found for *T* ≥ 25 °C, with ample changes corresponding to variations of few degrees, thus highlighting accurate temperature-sensitivity. At 35 °C, the transmittance of incident light for fabrics firstly programmed by 80 mJ/cm^2^ increases up to ΔTr ~ 30% (black dots in Fig. 1d), while the surface reflectance correspondingly decreases (open dots in Fig. 1d).

### Pattern activation: morphological and theoretical studies

**Fig. 2:**
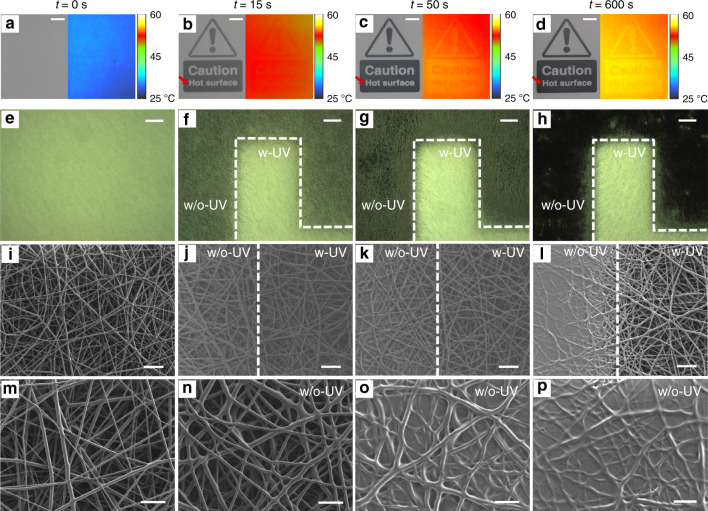
Pattern activation and changing morphology. **a** Photographs (left) and thermal images (right) of fibrous samples after the second step of photo-programming. **b**–**d** Photographs and thermal images of the sample in **a** captured at different times during heating at 55 °C. The color scale in thermal images indicates the temperature of the sample. (Scale bars in (**a**–**d)**: 5 mm). **e**–**h** Corresponding optical micrographs of **a**–**d** for a feature of the letter “H” in the sign “Hot” (red arrow in (**b**–**d**)). w-UV (w/o-UV) indicates the area of the sample exposed (unexposed) to UV light during the second step of photo-programming. Scale bars, 200 μm. **i**–**l** Corresponding SEM micrographs at the interface between the UV exposed (w-UV) and the unexposed areas (w/o-UV), collected at the various time points. Scale bars, 20 μm. **m**–**p** Higher magnification view of the areas shown in (**i**–**l**), that are not exposed to UV light during the second step of photo-programming. Scale bars, 10 μm.

An exemplary device displaying a warning sign upon exposure to a temperature of 55 °C is photographed at various time instants in the left images in Fig. 2a–d (an exemplary transition is also shown in the Supplementary Movie), and the corresponding thermal pictures collected by an infrared thermo-camera are shown in the right images. The visual output of the device is clearly based on the contrast in reflectance, which arises upon heating, between regions that were or were not previously exposed during the second photo-programming step, respectively. Exposed regions remain whitish, with high reflectance values, whereas unexposed areas that did not complete photo-induced cross-linking activation gradually melt into an almost continuous film and drastically decrease their reflectance, thus unveiling the substrate used for deposition. As such, the TTI exploits only ambient light with no need for powering or other light sources, and can display any desired shape or pattern depending on the optical mask used in the second photo-programming. Interestingly, the thermal images in Fig. 2b–d highlight that regions in which fibers are still present (i.e., where the negative SU-8 compound underwent the second photo-programming step) maintain a local temperature appreciably lower (by ∼1 °C, Fig. S8) than regions where fibers melt and a higher optical transmission is achieved, due to the cooling efficiency of the organic non-woven, which is likely to promote high heat fluxes^29^. The best view of the transition undergone by the non-woven fabric upon increasing temperature is captured by scanning electron microscopy (SEM) imaging of the interface across two regions that are either exposed or not exposed, during the second photo-programming step. Optical micrographs of this interface collected at different time points while the TTI is kept at 55 °C are shown in Fig. 2e–h, the corresponding SEM pictures of the fibrous interfaces are in Fig. 2i–l, and views at higher magnification of the unexposed fibers while melting over time are in Fig. 2m–p. As shown in Fig. 2i–l, fibers exposed during the second photo-programming are instead stable over the investigated times, which explains the different optical properties from a microscopic point of view.

**Fig. 3:**
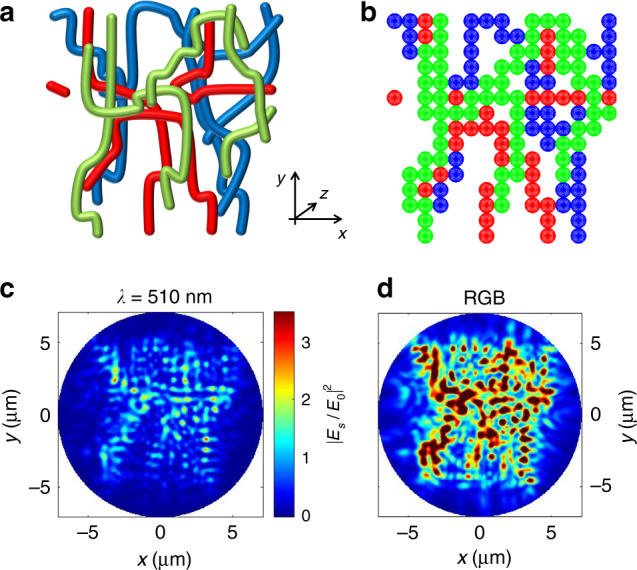
Light back-scattering from three-dimensional fiber non-wovens. **a** Scheme of the structure of nanofibers used for the calculations. To mimic the morphological structure of the fibrous non-woven, an area of 10 × 10 µm^2^ is considered in the *x*–*y* plane, and three layers of fibers are positioned along the axial (*z*) direction with total thickness 2.1 μm (green nanofibers are placed in the first layer, red ones in the second layer, and blue ones in the third layer). The average fiber diameter (700 nm) equals the value measured by SEM (Fig. S2, Supplementary Information). **b** Planar view (*x*–*y*) of cluster discretization used to calculate the light-scattering properties. Each polymer filament is schematized as a linear cluster with 700 nm spherical subunits. **c** Backscattering intensity map at *λ* = 510 nm, calculated as the ratio of the backscattered field (*E*_*s*_) and the incident one (*E*_0_). The map is obtained by considering a plane wave illumination with unitary amplitude impinging perpendicularly to the fibers (i.e., along the *z* axis), averaging over polarization, and it is calculated at about 1 μm distance from the first layer of the structure. **d** RGB intensity map obtained as the sum of the backscattering maps calculated at wavelengths corresponding to red (*λ* = 600 nm), green (530 nm,) and blue (450 nm) wavelengths.

To analyze this behavior in depth, we model the light-scattering properties of the fibrous non-woven fabrics by the transition matrix (T-matrix) technique^30–32^, schematizing each polymer filament as a linear aggregate of particle clusters with spherical subunits of 700 nm diameter (corresponding to the fiber average diameter). To mimic the random morphology of the fibrous surface, we consider three layers of fibers distributed along the thickness direction (*z* axis in Fig. 3a, b). The model structure is schematized in Fig. 3a, b, where green, red, and blue clusters represent filaments in the first, second, and third layer, respectively. The backscattering intensity map at *λ* = 510 nm (given by the squared ratio of the backscattered and the incident field, |*E*_*s*_/*E*_0_|^2^) is obtained by considering a plane-wave illumination with unitary amplitude impinging perpendicularly to the structure (i.e., along the *z* direction), averaging over polarization, and it is calculated at ∼1 μm distance from the first layer of the structure (Fig. 3c). A critically important parameter that yields quantitative information on the amount of the backscattered light is the albedo value, *A* = *C*_scat_/*C*_ext_, defined as the ratio of the scattering, *C*_scat_, and extinction, *C*_ext_, light-scattering cross-sections. T-matrix calculations yield an albedo of about *A*_510_ = 0.73 at *λ* = 510 nm, which is one order of magnitude higher than the reflectivity of a continuous film of SU-8 calculated from the Fresnel coefficients at normal incidence (0.06). To assess if this behavior has general validity for ambient light, we determine the backscattering maps at wavelengths corresponding to the RGB standard, 450, 530, and 600 nm (Fig. S9). In Fig. 3d we show the backscattering intensity map obtained as the sum of the single-wavelength components, evidencing the highly intense scattering occurring by ambient illumination that leads to an averaged albedo, *A*_RGB_ = 0.733. In addition, upon the fiber melting due to increasing temperature, we find that the albedo values dramatically decrease and approach those of SU-8 films. This investigation is based on a three-dimensional model taking into account a fibrous multi-layer, and it fully rationalizes the whitish appearance found for nanofiber non-wovens and the surfaces coated with them, as well as the reflectance contrast arising in our indicators during the operation.

T-matrix calculations also help in rationalizing the photon transport properties of the non-wovens, which are characterized by two length scales^33^. The first one is the scattering mean free path, *l*_s_, which is the distance of free propagation between scattering events^34^. This is calculated from the average cluster subunit density (*N* = 0.51 μm^−3^ for our clusters) and the spherical subunit scattering cross-section (σ_scat_ = 1.6 μm^2^ at *λ* = 510 nm), leading to *l*_s_ = 1/*N*σ_scat_ = 1.2 μm. The second relevant length scale is associated with the transport mean free path, l*, which is the distance over which the direction of propagation of the photon is randomized. This quantity is calculated by considering the subunit anisotropy parameter, *g* = *<*cos *θ>*, where *θ* is the scattering angle, and the similarity relation^33^, l* = *l*_s_/(1−*g*). For our structures we obtain *g* = 0.65 and a typical transport length of l* = 3.4 μm (see Supplementary Information for details). Thus, visible light impinging on the fibrous material is efficiently extinct within few microns of propagation. In addition, estimating the albedo values for samples with thickness matching experimental values (20–30 μm) shows how the intensity of the backscattered light is efficiently maximized with the used geometry (Fig. S10), thus leading to reflectance contrast in the indicators.

### Time–temperature indicator devices

Photo-programming through sequential exposures makes these devices highly versatile in terms of targeted time–temperature profiles, since calibrated doses tailored to the cross-linking degree and hence the structural relaxation of the non-wovens allow the optical response of the indicators to match the expiration intervals of very different perishable products (see Tables S1 and  S2 in the Supplementary Information) at a given temperature or through a varied thermal history. Examples to illustrate the ample range of times that need to be monitored include food, for which a few minutes up to hours at unsuitable temperature are enough to induce pathogen growth and toxin formation, drugs used by people traveling across different climatic zones (∼hours), and vaccines such those based on toxoids or polysaccharides that are found to degrade significantly in a few days just above the room temperature (35–45 °C)^6^. All these cases could be well covered by the thermal response of properly photo-programmed TTIs as shown in Fig. 1b. Strategies to further increase the operational timescale would include enhancing the thermal stability by calibrating the amount of photoacid generator in electrospun blends or tuning the substrate reflectivity properties, or electrospinning nanocomposites with slower relaxation dynamics, namely with reduced local mobility of filler-interacting polymer chains or cross-linked clusters, as demonstrated for other organic matrices^35^.

**Fig. 4:**
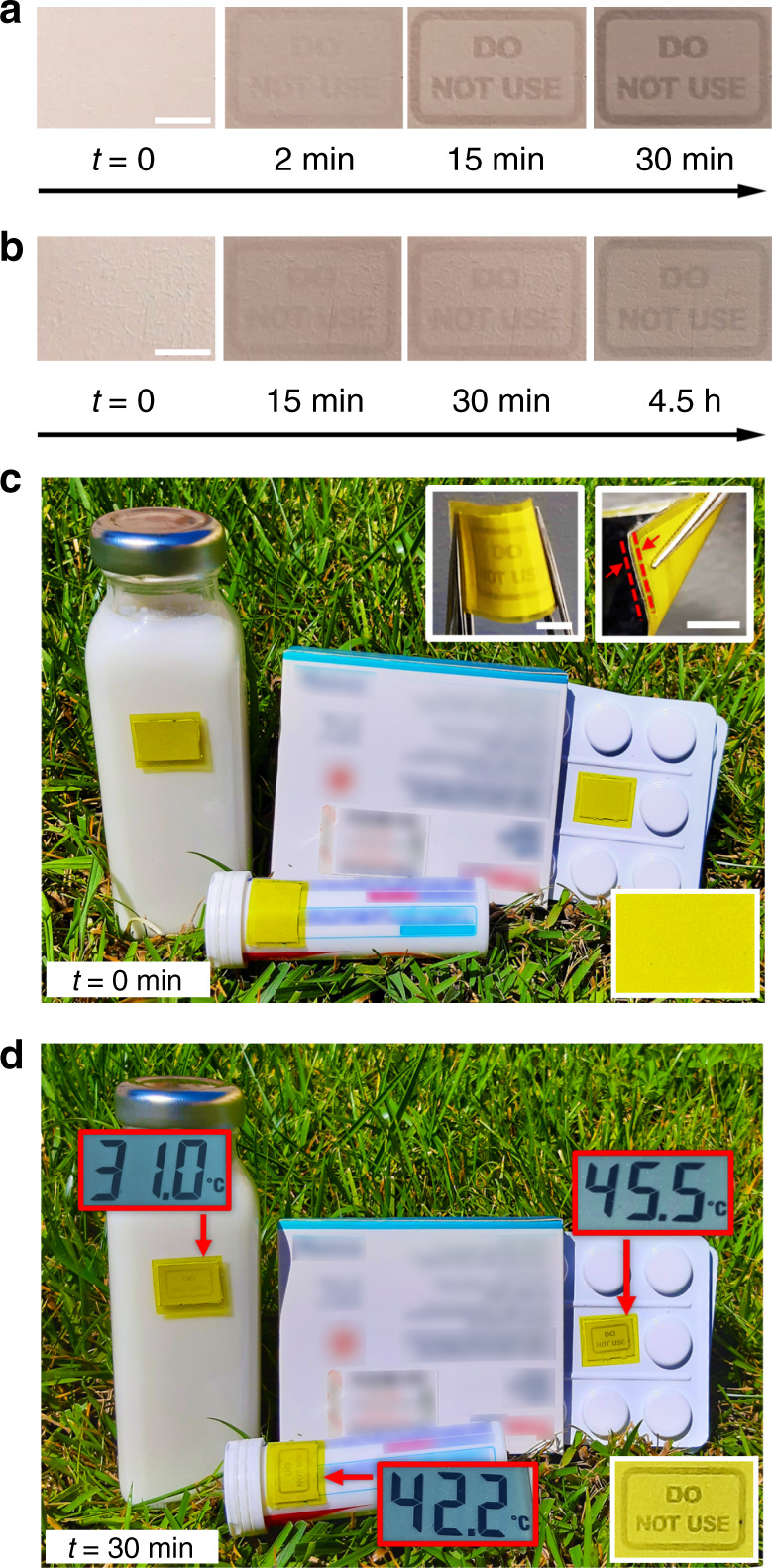
Flexible time–temperature indicator on paper substrate during operation. **a**, **b** Photographs of two differently programmed indicators while working at 35 °C with different response timescales: **a** minutes (device first photo-programming dose = 72 mJ/cm^2^) and **b** hours (first photo-programming dose = 80 mJ/cm^2^), captured at different observation times. (Scale bar: 5 mm). **c**, **d** Operation during sunlight-induced warming. Exemplary goods (milk, drugs) are exposed to sunlight for 30 min. The temperature of each item, measured by a thermocouple, is displayed in the red box. The photographs also show the corresponding visual response of non-wovens, encapsulated by yellow plastic sheets to screen UV radiation and prevent undesired residual crosslinking (**c**, top-right inset, scale bars, 5 mm). In the top-right inset in (**c**), arrows highlight the flexible and robust multilayer interface at the device edge. Bottom-right insets: magnified view of the indicator on the drug blister, before (**c**) and after (**d**) sunlight warming.

Exemplary visual sequences of two devices encoded by a first exposure step at 72 and 80 mJ/cm^2^, respectively, and then kept at 35 °C, are shown in Fig. 4a, b. Here, a “Do not use” sign appears while the reflectance contrast increases in timescales of minutes for the first devices and of hours for the second one. Higher or lower targeted temperature, as well as longer time response, can be achieved by controlling the cross-linking as shown in Fig. 1b. In addition, the devices can be made flexible and water-proof by directly depositing the fibers onto paper and encapsulating in commercial polyethylene terephthalate (PET) sheets (Figure S11), thus being conformable even to curved surfaces (Fig. 4c, inset). Yellow PET sheets can be also used for encapsulation, which allows the TTIs to operate outdoor under direct sun light as shown in Fig. 4c, d.

Furthermore, we point out that even changes in the device reflectance contrast that are too low to be appreciated by eye, could be easily detected by automatized time-lapse analysis of the Weber’s contrast (*C*_w_):

1 $$C_{\mathrm{w}} = \frac{{I_{\mathrm{b}} - I}}{{I_{\mathrm{b}}}}$$

where *I* is the mean gray intensity of the darker features and *I*_b_ is the background gray intensity in the activated pattern, respectively^36,37^. The typical temporal evolution of *C*_w_ exhibits a relatively rapid increase at *T*_w_, then approaching a saturation value (Fig. S12), similar to ΔTr data (black dots in Fig. 1d). A strategy for contrast enhancement is to vary the reflectivity properties of the deposition substrate. To this aim, TTIs were realized by using a glossy paper as substrate (Fig. S13a–d). A maximum value of *C*_w_ = 0.5 is found in this way (Fig. S13e, f) that is a factor 2.5 higher than the *C*_w_ measured for TTI devices with a similar characteristic timescale but made on opaque paper. Here, the dependence of the *C*_w_ on the thickness (*h*) of the non-woven can be rationalized considering the effective UV dose delivered to the regions that are exposed only during the first photo-programming step (corresponding to the dark regions of the patterns shown in Fig. S13a–d). For *h* < *h*_*M*_ (*h*_*M*_ = 30 µm, Fig. S13f), the UV intensity at the glossy paper substrate (*I*_s_^UV^) is higher than for a sample with thickness *h*_*M*_: *I*_s_^UV^(*h* < *h*_*M*_) > *I*_s_^UV^(*h* = *h*_*M*_). Due to the diffuse reflectivity of the glossy paper (∼5%), a non-woven with *h* < *h*_*M*_ thus receives an effective higher UV dose with respect to the one with *h* = *h*_*M*_, leading to relatively higher SU-8 crosslinking and improved capability to retain the filamentary structure at the programmed working temperature. This in turn enhances the intensity of the light that is backscattered from these regions, and relatively decreases the pattern contrast. While the intensity at the glossy paper also decreases for thicker non-woven [*I*_s_^UV^(*h* = *h*_*M*_) > *I*_s_^UV^(*h* > *h*_*M*_)], for such samples an additional effect becomes more relevant. Indeed, in this case additional UV exposure of the regions that have undergone the first photo-programming step can occur during the second programming step, because the scattering of UV light through the non-woven can determine a lateral spread of the illuminated features. This effect becomes more relevant upon increasing *h*, leading to the data curve plot in Fig. S13f. Finally, we carry out long-term stability experiments on the devices to determine the most appropriate storage temperature prior use, finding that freezing conditions are suggested to assure a month-scale shelf-life for these indicators (Figs. S14 and S15). Further studies are currently underway to further extend storage limits.

## Discussion

Collectively, these results showed that the thermal response of materials for an intelligent indicator can be engineered by tuning the degree of cross-linking and translating to nano-textured non-wovens the working principle of negative resists commonly used in micro- and nano-lithographies. At working temperature, changes take place that alter the material architecture, and consequently, the optical behavior. Therefore, the obtained devices are very cheap, flexible, and light-weight, compatible with paper and common layer encapsulation, and conformable to monitored items with any size and shape. The operation simply consists of visual testing, with no need for electronics or moving/liquid component, and is explained by backscattering intensity from the complex fibrous material, here analyzed though three-dimensional calculations. Outperforming existing solutions in terms of programming capability and ease of use, nano-materials with photo-encoded thermal properties have established the design rule for intelligent labels for the supply chain of perishable products.

## Methods

### Photo-programmed nanofibers

SU-8 3025 (Microchem, product Y3110720500L1GL, lot number 18030250) was diluted with acetone (Sigma) at different relative volumes (acetone/SU-8 volume) in the range 0–16% to form test solutions for electrospinning. Uniform and smooth fibers (Fig. S1) with an average diameter of 700 nm (Fig. S2) were obtained with 9% relative acetone/SU-8 volume and an applied electric field of 0.7 kV/cm, producing electrified jets from a 27-gauge stainless-steel needle. Electrospinning times between 7 and 13 min led to non-wovens with thickness between 20 and 30 μm, measured by profilometry (DektakXT, Bruker). Substrates used to successfully deposit the fibers were silicon wafers, aluminum foils, indium tin oxide-coated glass, and paper. The first step of photo-programming was carried out by UV exposure (*λ*_max_ ∼ 365 nm) from cylindrical lamps at a distance of 10 cm from the sample, with doses in the range 0.3–80 mJ/cm^2^. The second, spatially selective step of photo-programming (pattern activation, 250, 305 mJ/cm^2^) was carried out by a MJB4 (SUSS MicroTec) mask aligner system or by a low-cost bromograph. The doses of UV exposure were measured by using a calibrated power meter (mod. 843-R, Newport) equipped with a UV-visible detector (mod. 818-UV/DB, Newport). More specifically, the detector was positioned in place of the sample and the UV intensity measured as a function of time with a temporal resolution of 0.5 s. The effective UV dose impinging on the samples was then calculated by integrating the measured temporal curve of the UV intensity in a time interval corresponding to the various exposure times used in the photo-programming steps. Packaging of flexible indicators was performed by embedding the non-woven within two plastic foils with a thickness of 75 μm, through a commercial cold laminating machine (Speedy A4, Buffetti).

### Thermal, optical, and morphological properties

DSC was performed by a Q200 differential scanning calorimeter from TA Instruments-Waters LLC, equipped with a RCS90 cooling system and a nitrogen gas purge set at a flow rate of 50 mL/min. Standard sample pans in aluminum were used and sealed before measurements. Non-woven samples with mass in the range 2.5–4.3 mg were heated from −60 to 250 °C at 10 °C/min. IR spectroscopy was performed with a FTIR spectrophotometer (Spectrum 100, Perkin-Elmer Inc.) in transmission mode, measuring at least three different samples for each dose. Thermograms were analyzed by TA Universal analysis. The optical transmission of non-wovens was measured by an UV/VIS spectrophotomer Lambda 950 (Pelkin Elmer). The FEG-SEM Merlin from Zeiss at acceleration voltages of 1–18 kV was used to image fibers deposited on silicon substrates. The diffuse reflectance was measured by using a broadband lamp^38^ as light source and an integration sphere (Labsphere) coupled to a spectrometer through an optical fiber (mod. Flame, Ocean Optics). The sample was positioned in place of the access port of the sphere, which is collinear with the one used for the incident light beam. The incident light beam was directed onto the sample along the direction perpendicular to the sample surface, and only the hemispherically diffused light was detected by the sphere and spectrometer. The data of light backscattered by the sample (along the direction of the incident beam) at a solid angle of 1.1 × 10^−3^ sr was not collected. As a reference sample, we used a blank port coated with the same high diffusive layer of the internal surface of the integration sphere. An infrared camera (FLIR A655sc with a macroscopic lens) was used to monitor the temperature of the sample during heating at 55 °C for 10 min.

### Modeling

To calculate the maps of the backscattered intensity and the albedo, *A*, of the model non-woven, light scattering theory in the T-matrix formalism was exploited^30–32^ as generalized in the cluster model^39,40^ (see Supplementary Information). Organic filaments are modeled as a one-dimensional cluster of spherical particles with diameter equal to the average nanofiber transverse size. The structure of the network is constructed in three dimensions to mimic a 10 × 10 µm^2^ area of the nanofiber mats with a thickness of 2.1 µm. The backscattering intensity maps are calculated as the ratio |*E*_*s*_/*E*_0_|^2^, where *E*_*s*_ and *E*_0_ are the amplitude of the backscattered and incident fields, respectively, considering a plane wave illumination and averaging over polarization states.

### Weber’s contrast

The temporal evolution of *C*_w_ was evaluated on devices prepared by using the following procedure: (i) fiber deposition on a paper substrate, (ii) first step of photo-programming (72–80 mJ/cm^2^), (iii) second step of photo-programming (305 mJ/cm^2^), and (iv) device encapsulation by cold lamination between PET sheets. Images of the TTIs maintained at a constant temperature of 35 °C were measured at several time points using either a smartphone photo-camera or an imaging system based on a CMOS camera (DCC1645C, Thorlabs) coupled with a long working-distance optical system (MVL7000, Thorlabs). For the calculation of *C*_w_ the images of the sample were converted to 8-bit and the mean gray intensities of the exposed and unexposed areas were estimated by using the Fiji-ImageJ software (U.S. National Institutes of Health, Bethesda, Fig. S12 and S13).

## Supplementary information

Supplementary Information

Description of Additional Supplementary Files

Supplementary Movie 1

## Data Availability

Data in support of the findings of this study are available from the corresponding authors upon reasonable request.

## Code availability

Codes in support of the findings of this study are available from the corresponding authors upon reasonable request.
