# Supplementary material for: Intelligent non-colorimetric indicators for the perishable supply chain by non-wovens with photo-programmed thermal response
Source: Nat Commun. 2020 Nov 25;11:5991. doi: 10.1038/s41467-020-19676-y (PMC7688929; doi:10.1038/s41467-020-19676-y)
Supplement: Supplementary file 1 — Supplementary Information [file 41467_2020_19676_MOESM1_ESM.pdf]

Intelligent non-colorimetric indicators for the perishable supply chain by non-wovens with photo-programmed thermal response

Luigi Romano[1,*], *Alberto Portone*[1,*], *Maria-Beatrice Coltelli*[2], *Francesco Patti*[3,4], *Rosalba Saija*[3,4], *Maria Antonia Iatì*[4], *Giuseppe Gallone*[2], *Andrea Lazzeri*[2], *Serena Danti*[2,5], *Onofrio M. Maragò*[4], *Andrea Camposeo*[1], *Dario Pisignano*[1,6,§] *and Luana Persano*[1,§]

[1] NEST, Istituto Nanoscienze-CNR and Scuola Normale Superiore, Piazza San Silvestro 12, I-56127 Pisa, Italy
[§] E-mail: luana.persano@nano.cnr.it

[2] Department of Civil and Industrial Engineering, University of Pisa, Largo L. Lazzarino 2, I-56122 Pisa, Italy

[3] Dipartimento di Scienze Matematiche e Informatiche, Scienze Fisiche e Scienze della Terra, Università di Messina, Viale F. Stagno d'Alcontres 31, I-98166 Messina, Italy

[4] CNR-IPCF, Istituto Processi Chimico-Fisici, Viale F. Stagno D'Alcontres, 37, I-98158 Messina, Italy

[5] Department of Civil and Environmental Engineering, Massachusetts Institute of Technology, 33 Massachusetts Ave, Cambridge, MA 02142, USA

[6] Dipartimento di Fisica, Università di Pisa, Largo B. Pontecorvo 3, I-56127 Pisa, Italy
[§] E-mail: dario.pisignano@unipi.it

[*] These authors contributed equally to this work

SUPPLEMENTARY INFORMATION

Supplementary Figures

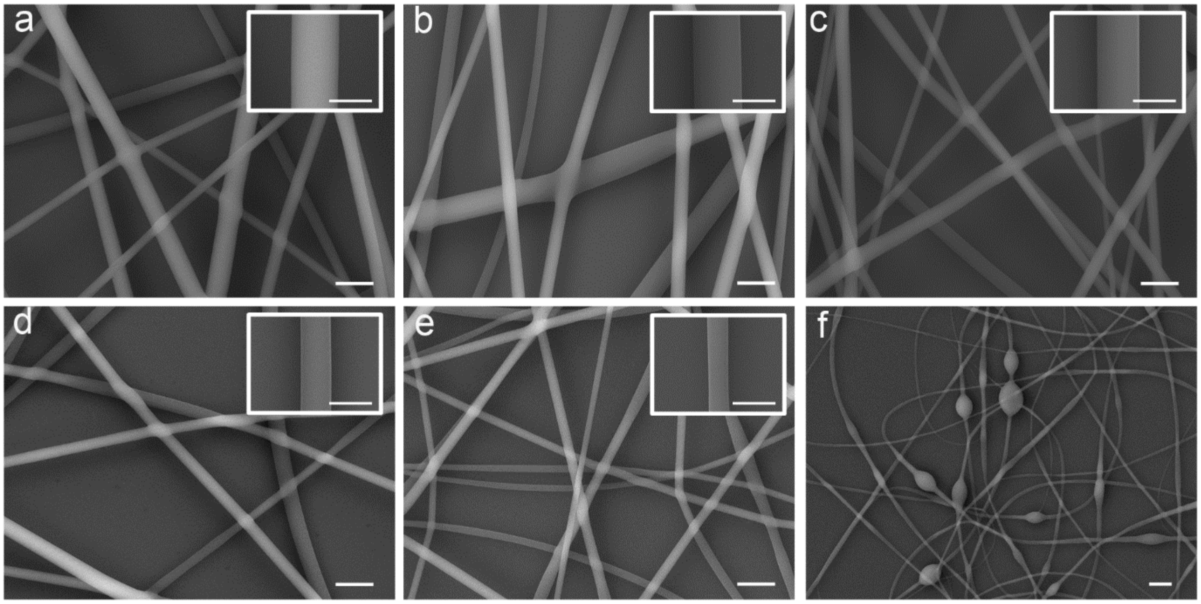

Figure S1 | SEM micrographs of SU-8 fibers spun upon dilution in acetone at different relative volumes (acetone/SU-8 volume): **a**, 0%, **b**, 3%, **c**, 7%, **d**, 9%, **e**, 12% and **f**, 16%. Additional electrospinning parameters: flow rate= 1 mL/h, needle-to-collector distance= 30 cm, electric field= 0.7 kV/cm. Scale bars, 2 µm (**a**-**f**) and 1 µm (insets **a**-**e**).

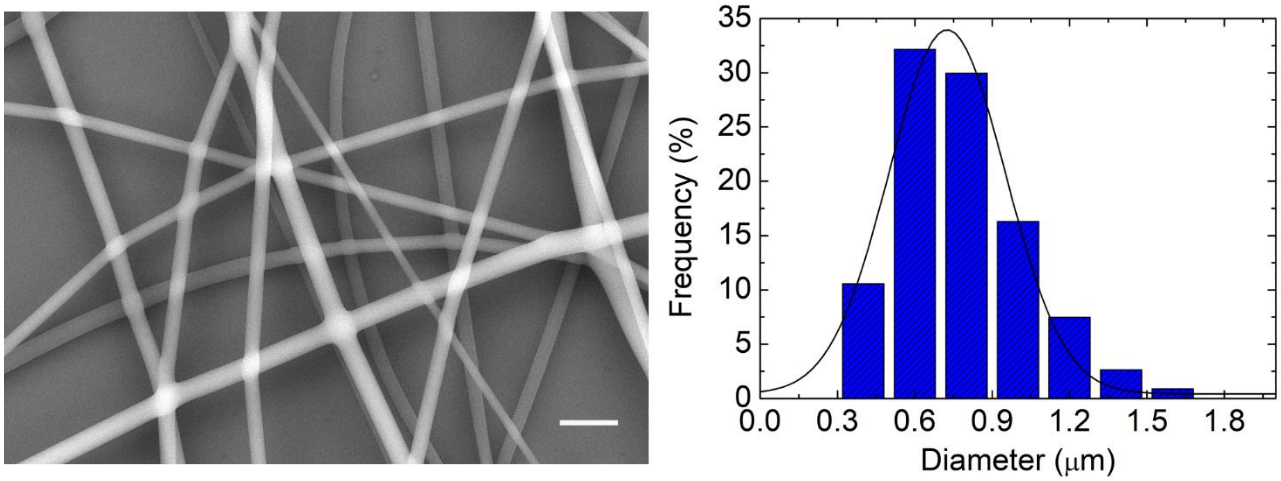

Figure S2 | Exemplary SEM micrograph (left, scale bar: 2 µm) and statistical distribution (right) of the diameters of SU-8 fibers spun upon dilution in acetone (9% acetone/SU-8 volume). Additional electrospinning parameters: flow rate= 1 mL/h, needle-to-collector distance= 30 cm, electric field= 0.7 kV/cm. The statistical distribution is calculated over 227 fibers imaged by SEM in different areas of the same sample.

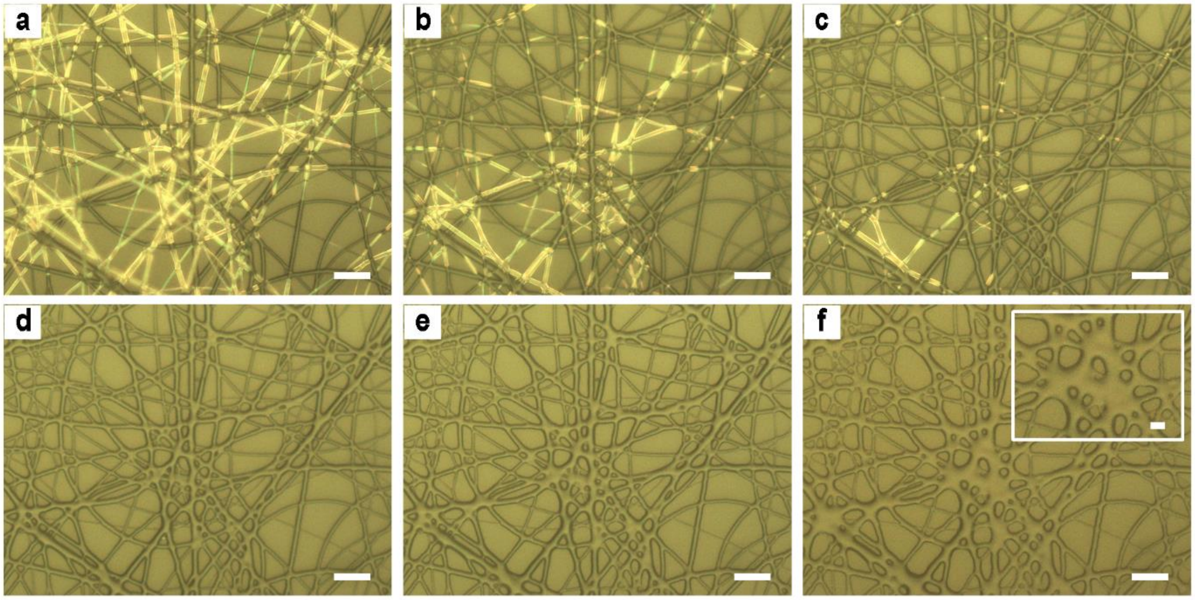

Figure S3 | Optical micrographs of unprogrammed (pristine) SU-8 fibers, kept at room temperature for different time durations: **a**, 0 **b**, 0.5 h, **c**, 1 h, **d**, 5 h, **e**, 9 h and **f**, 26 h. Electrospinning parameters: acetone/SU-8 volume= 9%, flow rate= 1 mL/h, needle-to-collector distance= 30 cm, electric field= 0.7 kV/cm. The deposition time is 2 minutes. Scale bar, 10 µm. Inset: magnification view of **f**, scale bar, 3 µm.

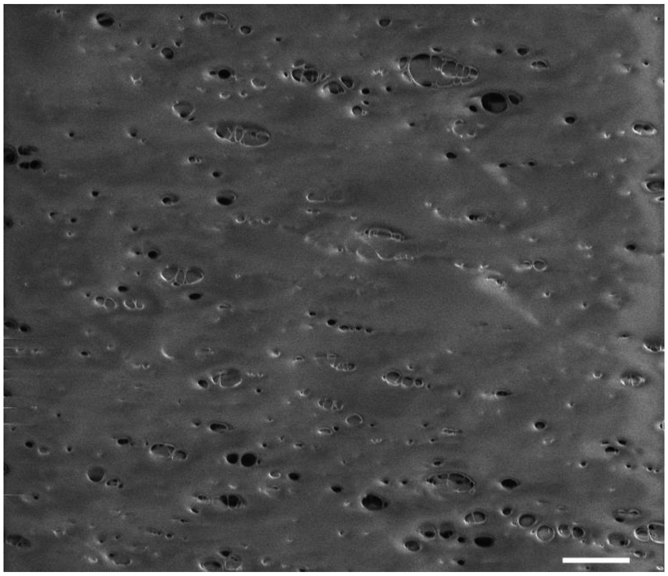

Figure S4 | SEM micrograph of the film resulting from unprogrammed (pristine) SU-8 fibers kept at room temperature for one night. Scale bar, 25 μm.

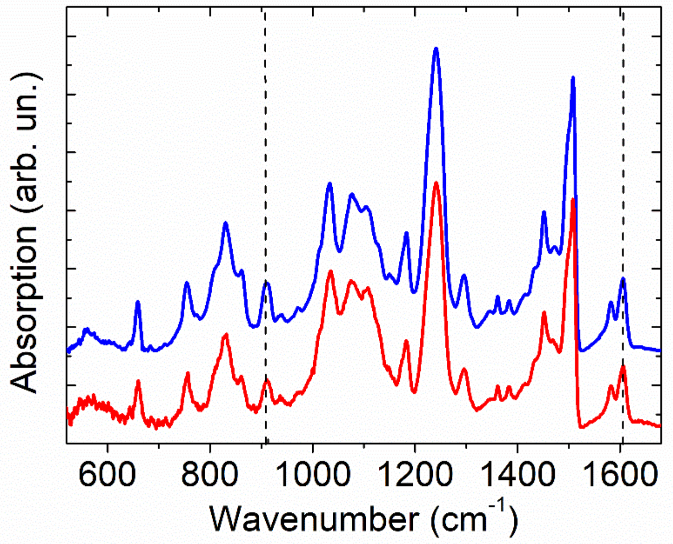

Figure S5 | FTIR spectra measured on SU-8 fibers. Blue line: as-spun fibers. Red line: fibers at the second step of photo-programming by an exposure dose of 305 mJ/cm^2. The dashed lines indicates the peak at 911 cm^{-1}, assigned to epoxy ring vibration, and the reference peak at 1607 cm^{-1}, used to calculate the cross-linking degree, Γ.

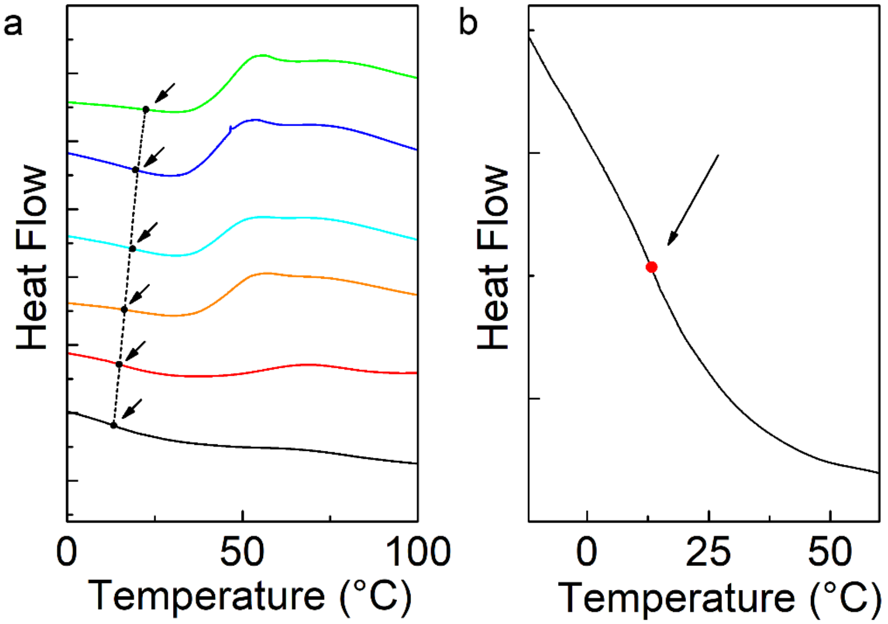

Figure S6 | a, DSC thermal analysis of unprogrammed (pristine) nano-textured SU-8 non-wovens (bottom curve), and (from bottom to top) for non-wovens exposed by 0.3, 23, 43, 62, and 80 mJ/cm^2 (first photo-programming step). Regions of DSC profiles with inflection points (dots) indicative of T_g gradually move to higher temperature (dashed line, guide for the eye) upon increasing the exposure dose. Exothermic peaks are visible at temperature ≥ 50°C, possibly partially hindering the glass-transition behaviour for high exposure dose. The exothermic peaks are associated with polymerization induced by a very low amount of active initiator [1] and with thermal curing of the organic system [2]. **b**, DSC magnification of the glass transition temperature region for the unprogrammed sample. T_{g0} ≅13°C (highlighted by the arrow).

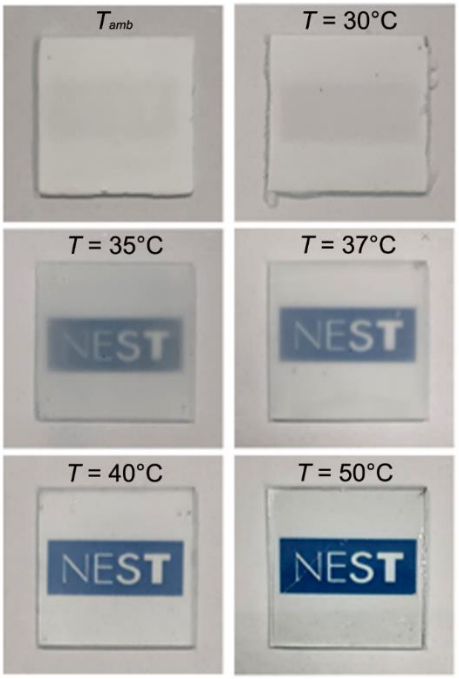

Figure S7 | Photographs of SU-8 non-wovens, deposited onto glass substrates covered with indium tin oxide and kept at different temperatures for 60 minutes. First photo-programming dose = 80 mJ/cm^2. T_{amb}: ambient temperature, about 20°C. The logo "NEST" behind the samples becomes visible once upon temperature increasing and closely approaching T_w=38°C.

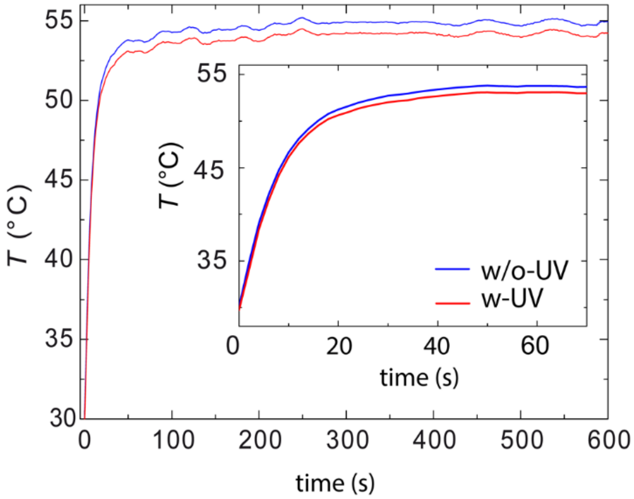

Figure S8 | Quantitative analysis of the thermal gradient between sample regions unexposed (w/o-UV) and exposed (w-UV) to UV light during the second step of photo-programming (as in Figure 2a-d). First photo-programming dose = 80 mJ/cm^2. Second, spatially-selective photo-programming dose ≅ 250 mJ/cm^2. Device operating at 55 °C. Inset: magnification view (interval 1-60 s).

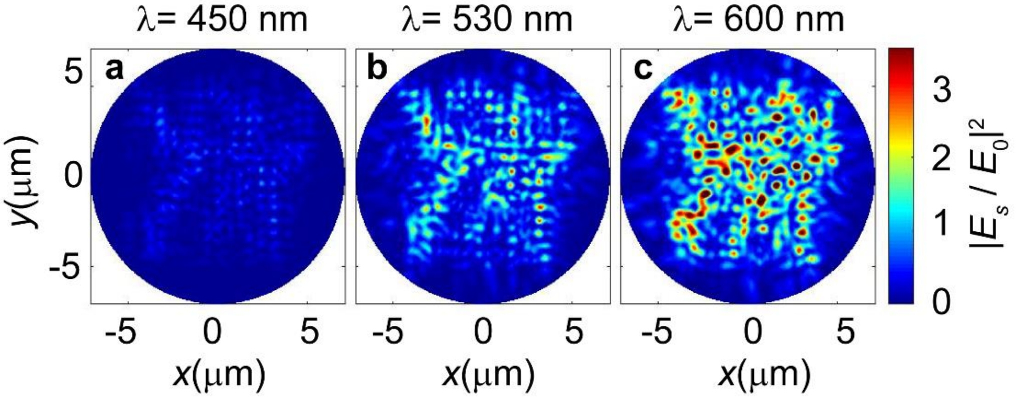

Figure S9 | Backscattering intensity maps at RGB reference wavelengths (450 nm, 530 nm, 600 nm). The field intensity maps are calculated by considering a plane wave illumination with unitary amplitude impinging perpendicularly to the structure, as in Figure 3. The maps are obtained averaging over polarization states and are calculated at about 1 μm distance from the first layer of the structure. The corresponding albedo value for the three different wavelengths is A_{450}=0.69, A_{530}=0.74, A_{600}=0.77.

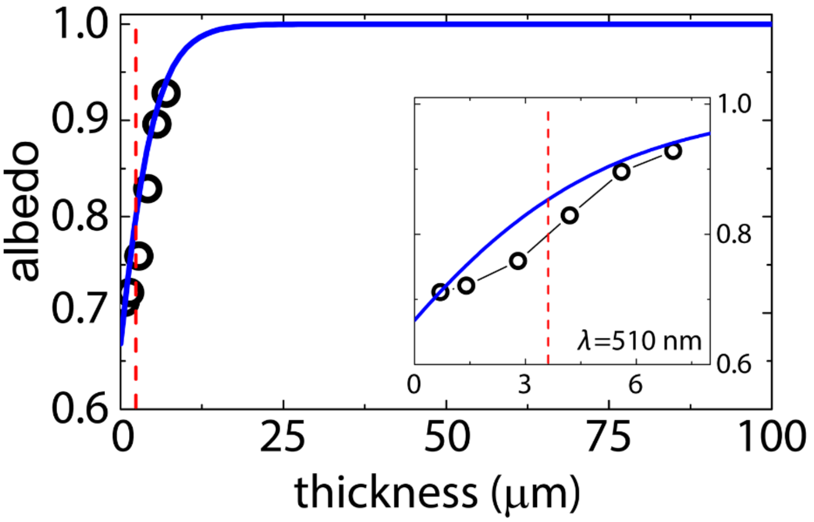

Figure S10. Albedo calculated by T-matrix formalism (circles) at λ = 510 nm, as a function of the non-woven thickness. The albedo is calculated for a system thickness = 700 nm, 1.4 μm, 2.8 μm, 4.2 μm, 5.6 μm, and 7.0 μm (namely up to ten layers). For non-absorbing structures, the albedo approaches the unity asymptotic value for thickness larger than the transport length (l*=3.4 μm, dashed red lines) calculated from the similarity relation (see details below). Blue line: a simple model of the albedo based on the Lambert-Beer extinction decay, with l*=3.4 μm and the albedo for the first layer is fixed at the value calculated by T-matrix (see details below). The inset is a close-up at small thickness, that shows the good agreement of the simple model to the albedo values calculated with T-matrix.

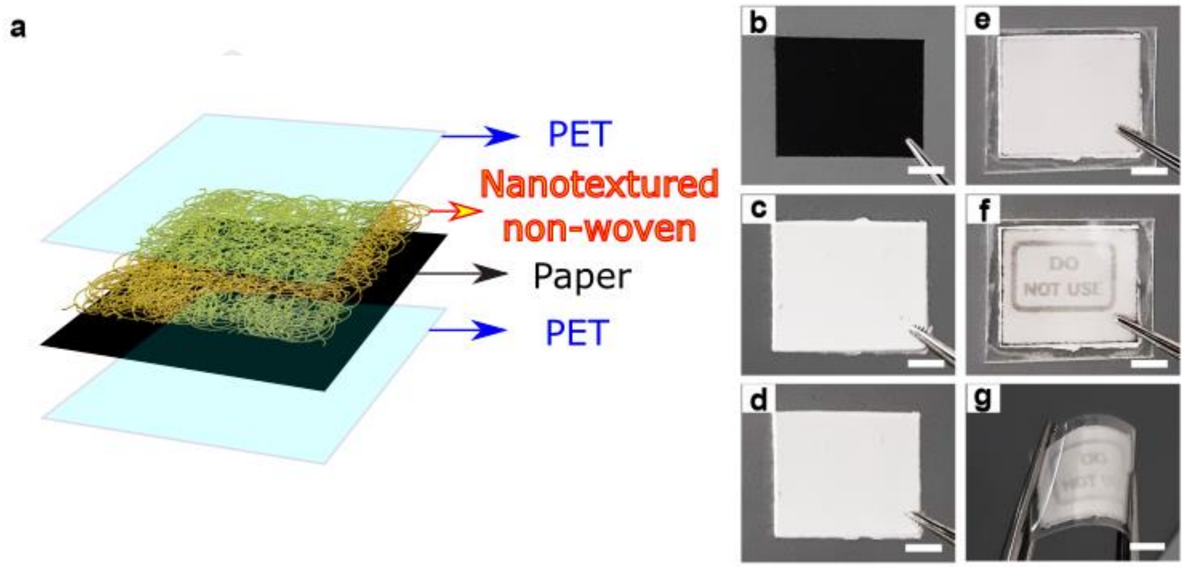

Figure S11 | a, Schematic representation of the assembly of a device consisting of a PET-encapsulated fibrous non-woven on paper. **b-e**. Representative photographs of the device at each fabrication step: **b**, black paper substrate; **c**, fiber deposition; **d**, UV photo-programming (step 1, dose: 72 mJ/cm^2 and step 2, dose: 305 mJ/cm^2); **e**. encapsulation with PET foils. **f**,**g**. Photographs of the device after thermal exposure at T= 55°C under static and bending conditions. Scale bar, 5 mm.

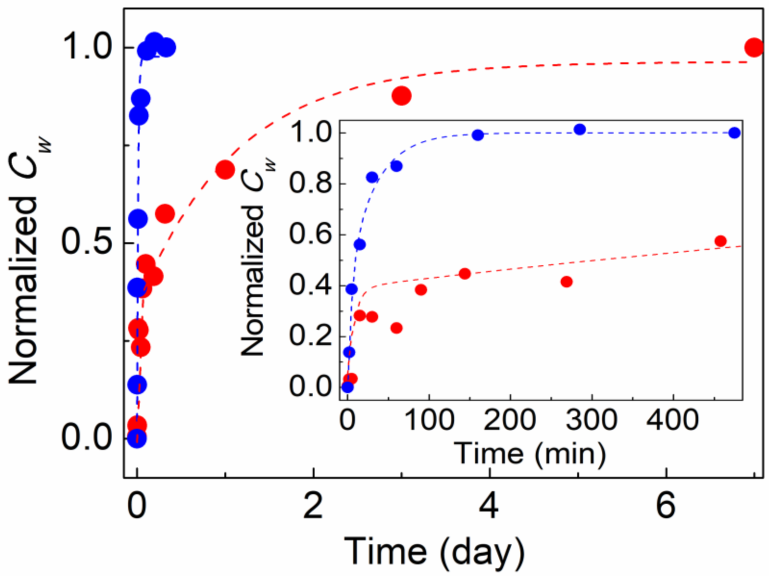

Figure S12 | Temporal evolution of the Weber contrast (C_w) in two differently-programmed indicators. First photo-programming dose: 72 mJ/cm^2 (blue dots) and 80 mJ/cm^2 (red dots). The devices operate at 35°C. Dotted lines are guides for the eye. Inset: magnification of the evolution of C_w in the first 8 h.

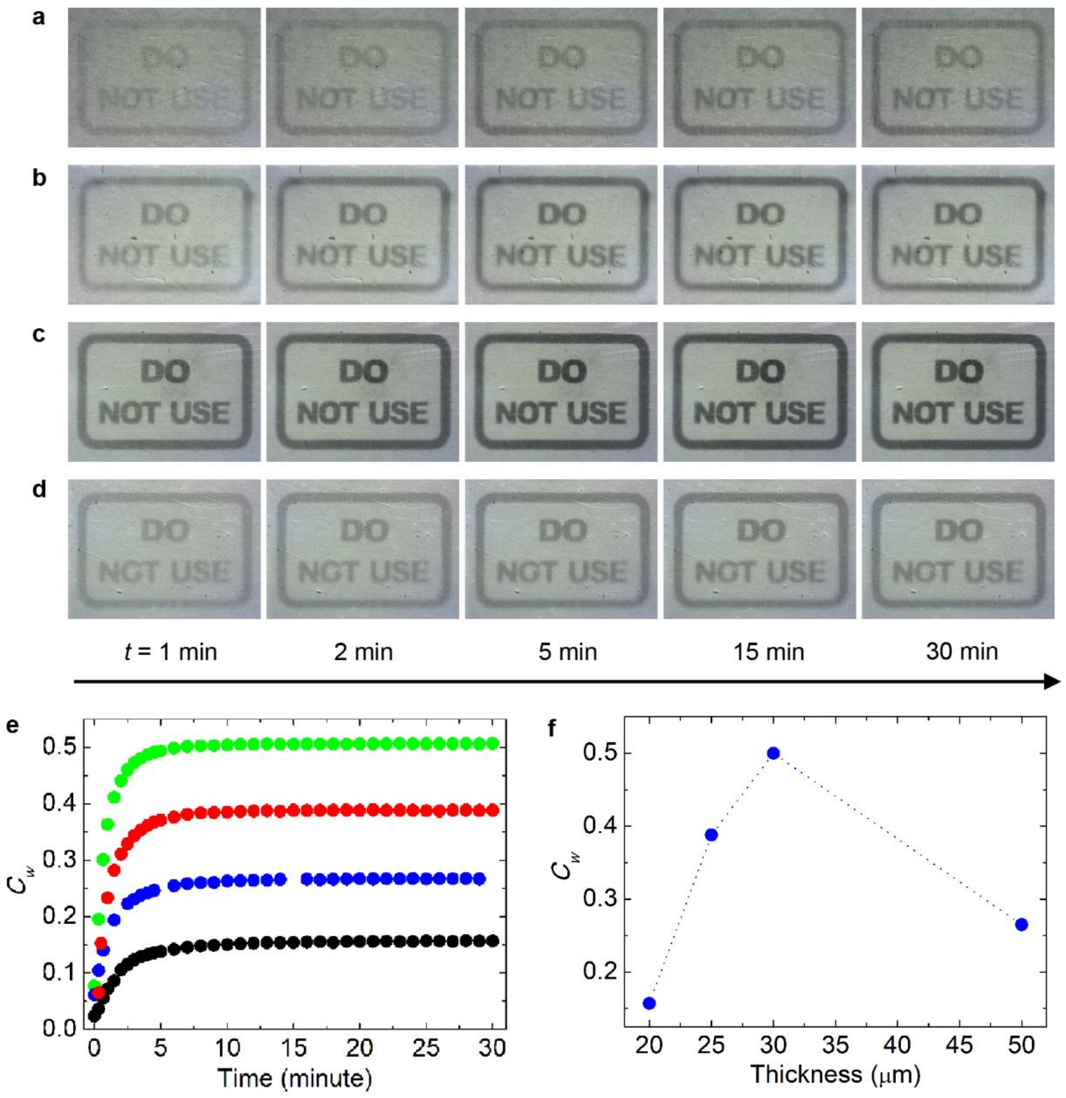

Figure S13. Flexible TTIs on glossy paper. **a-d**. Photographs of indicators while working at 35°C with response timescale of minutes (device first photo-programming dose = 72 mJ cm^{-2}) captured at different observation times. The indicators shown in **a-d** are realized with a thickness of the non-woven of: **a**, 20 µm; **b**, 25 µm; **c**, 30 µm; **d**, 50 µm. **e**, Temporal evolution of the Weber contrast (C_w) for samples with thickness of the non-woven of 20 µm (black circles), 25 µm (red circles), 30 µm (green circles), 50 µm (blue circles). **f**. Dependence of C_w on the thickness of the non-woven. C_w is calculated at t=30 minutes.

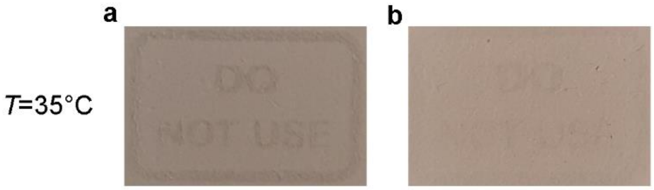

Figure S14 | Device operation upon storage at 24°C in dark prior use. Photographs of indicators upon heating at 35°C for 30 min. First photo-programming dose = 72 mJ/cm^2. Second, spatially-selective photo-programming dose = 305 mJ/cm^2. The storage time is: **a**, 7 days and **b**, 30 days. The device function upon storage at room temperature is largely degraded due to irreversible fiber cross-linking.

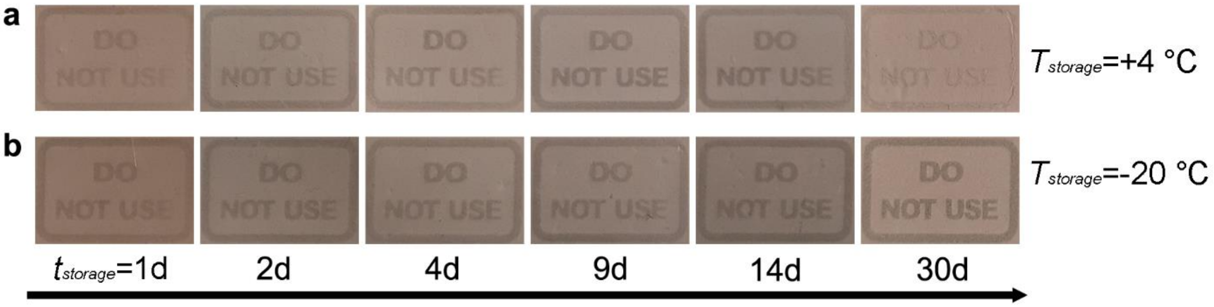

Figure S15 | Device operation upon storage at a temperature (*$T_{storage}$*) of +4°C and at -20°C in dark prior use. Photographs of indicators upon heating at 35 °C for 30 min. First photo-programming dose = 72 mJ/cm^2. Second, spatially-selective photo-programming dose = 305 mJ/cm^2. The corresponding storage time ($t_{storage}$) is reported below each photograph. The device function upon storage at +4°C and at -20°C is well-preserved.

Supplementary Note

Light scattering in the T-matrix formalism

Light scattering calculations are carried out by considering a monochromatic incident polarized plane wave described by the complex amplitude:

$$\boldsymbol{E}_I = E_0 \hat{\mathbf{e}}_I exp(ik_I \cdot r), \tag{S1}$$

with unit polarization vector $\hat{\mathbf{e}}_I$and propagation vector $\mathbf{k}_I = k\hat{\mathbf{k}}_I$, where $k=nk_v$ and $k_v=\omega/c$. Here, n is the refractive index of the external medium that is fixed to $n=1$ since the fibers are in air. The total field outside the particle is the sum of the incident and scattered field. The scattered field is calculated from the equations obtained by applying the customary boundary conditions across the particle surface linking the internal and external fields. The overall scattering problem can be solved by the transition matrix (T-matrix) method [3-8], based on the definition of a linear operator relating the incident field to the scattered field [3].

The starting point of the method is the field expansion in terms of vector spherical harmonics (VSH), i.e., the vector solutions of the Maxwell equations in a homogeneous medium that are simultaneous eigenfunctions of the angular momentum and the parity operators. The multipole expansion of the plane wave electric field and of the corresponding scattered wave is [4]:

$$\mathbf{E}_I = E_0 \sum_{plm} \mathbf{J}_{lm}^{(p)}(\mathbf{r},\mathbf{k}) W_{lm}^{(p)}(\hat{\mathbf{e}}_I, \hat{\mathbf{k}}_I) \tag{S2a}$$
$$\mathbf{E}_S = E_0 \sum_{plm} \mathbf{H}_{lm}^{(p)}(\mathbf{r},\mathbf{k}) A_{lm}^{(p)}(\hat{\mathbf{e}}_I, \hat{\mathbf{k}}_I) \tag{S2b}$$

where $\mathbf{J}_{lm}^{(p)}(\mathbf{r},\mathbf{k})$ denotes vector multipole fields that are regular at the origin of the coordinate system fixed inside the particle (Bessel multipoles), superscript $p=1,2$ refers to the values of the parity index, distinguishing magnetic multipoles ($p=1$) from electric ones ($p=2$), the $\mathbf{H}_{lm}^{(p)}$ denotes multipole fields which satisfy the radiation condition at infinity (Hankel multipoles) and they are identical to the $\mathbf{J}_{lm}^{(p)}$ multipole fields except for the substitution of the Hankel function of the first kind $h_l(kr)$ in place of the spherical Bessel functions $j_l(kr)$.

The scattering process can be fully described by a linear operator S defined by the equation:

$$\mathbf{E}_S = S\mathbf{E}_I \tag{S3}$$

The operator S (transition operator) can be introduced because of the linearity of the Maxwell equations and of the equations expressing the boundary conditions across the surface of the particle. The S representation on the basis of the VSH gives the T-matrix defined by the equation [3-6]:

$$A_{lm}^{(p)} = \sum_{p'l'm'} S_{lml'm'}^{(pp')}(r,k) W_{l'm'}^{(p')}(\hat{\mathbf{e}}_I, \hat{\mathbf{k}}_I) \tag{S4}$$

relating the multipole amplitudes of the incident field, $W_{l'm'}^{(p')}(\hat{\mathbf{e}}_I, \hat{\mathbf{k}}_I)$, to those of the scattered field, $A_{lm}^{(p)}$. The elements of the T-matrix, $S_{lml'm'}^{(pp')}(r,k)$, contain all the information about the scattering process, but are independent of the state of polarization of the incident field. The transformation properties of the multipole fields under rotation of the coordinate frame imply corresponding transformation properties of

the T-matrix under rotation of the scattering particle [8]. This is applicable to the calculation of the orientational average of all the quantities of interest for any choice of the orientational distribution function of the particles.

Aggregates of spheres. We modeled the backscattering properties of nanofiber non-wovens with specific thickness considering each fiber composed of several spherical particles and exploiting the T-matrix of such aggregate of spheres. In fact, the T-matrix approach solves the scattering problem by an aggregate (cluster) of spheres without resorting to any approximation [9-12].

In particular, we consider a cluster of spheres numbered by the index α, of radius ρ_α, and refractive index n_α, whose centers lie at $\mathbf{R}_\alpha$. The spheres may have, in principle, different refractive index and different radii. The field scattered by the whole aggregate is written as the superposition of the fields scattered by the single spheres:

$$\mathbf{E}_{S\eta} = E_0 \sum_\alpha \sum_{plm} \mathbf{H}_{lm}^{(p)}(r_\alpha, k) A_{\eta\alpha lm}^{(p)} \tag{S5}$$

where $\mathbf{r}_\alpha = \mathbf{r} - \mathbf{R}_\alpha$. The field within each sphere is taken in the form:

$$\mathbf{E}_{T\eta\alpha} = E_0 \sum_{plm} \mathbf{J}_{lm}^{(p)}(r_\alpha, k) C_{\eta\alpha lm}^{(p)} \tag{S6}$$

with $k_\alpha = n_\alpha k_v$, so that it is regular everywhere inside the sphere. The amplitudes $A_{\eta\alpha lm}^{(p)}$ and $C_{\eta\alpha lm}^{(p)}$ are determined by applying the boundary conditions tot he fields across the surface of each sphere. Thus, the scattered field is given by a linear combination of multipole fields with different origins, whereas the incident field is given by a combination of multipole fields centered at the origin of the coordinates. In order to write the whole field in terms of multipole fields centered at $\mathbf{R}_\alpha$ we use the addition theorem [4]. In this way the scattered field at the surface of the α-th sphere turns out to be [4,12]:

$$\mathbf{E}_{S\eta} = E_0 \left[\sum_{plm} \mathbf{H}_{lm}^{(p)}(\mathbf{r}_\alpha, k) A_{\eta\alpha lm}^{(p)} + \sum_{\alpha'} \sum_{p'l'm'} \mathbf{J}_{lm}^{(p)}(r_\alpha, k) H_{\alpha lm\,\alpha' l' m'}^{(pp')} A_{\eta\alpha' l' m'}^{(p)}\right] \tag{S7}$$

where the operator *H* is a transfer matrix. Analogously, the incident field at the surface of the α-th sphere is [4,12]:

$$\mathbf{E}_{I\eta} = E_0 \sum_{plm} \sum_{p'l'm'} \mathbf{J}_{lm}^{(p)}(\mathbf{r}_\alpha, k) J_{\alpha lm\,0l'm'}^{(pp')} W_{I\eta l'm'}^{(p')} \tag{S8}$$

where $\mathbf{R}_0 = 0$ is the vector position of the origin. Applying the boundary conditions, we get for each α four equations among which the elimination of the amplitudes of the internal field can be applied. In this way we get a system of linear inhomogeneous equations:

$$\sum_{\alpha'} \sum_{p'l'm'} M_{\alpha lm\,\alpha' l' m'}^{(pp')} A_{\eta\alpha lm}^{(p)} = -\mathcal{W}_{I\eta\alpha lm}^{(p)}, \tag{S9}$$

where we define:

$$\mathcal{W}_{I\eta\alpha lm}^{(p)} = \sum_{p'l'm'} J_{\alpha lm\,0l'm'}^{(pp')} W_{I\eta\alpha lm}^{(p')} \tag{S10a}$$

and

$$M^{(pp')}_{\alpha lm\,\alpha' l' m'} = (R^{(p)}_{\alpha l})^{-1}\delta_{\alpha\alpha'}\delta_{pp'}\delta_{ll'}\delta_{mm'} + H^{(pp')}_{\alpha lm\,\alpha' l' m'} \quad \text{(S10b)}$$

The quantities $R^{(p)}_{\alpha l}$ are the Mie coefficients for the α-th sphere. The quantities $H^{(pp')}_{\alpha lm\,\alpha' l' m'}$ which result from the addition theorem, describe the multiple scattering processes occurring among the spheres in the aggregate. The occurrence of these processes is indicative of inter-spheres coupling. The elements $H^{(pp')}_{\alpha lm\,\alpha' l' m'}$ of the transfer matrix couple multipole fields both of the same and of different parity, with origin on different spheres. The formal solution to system (S9):

$$A^{(p)}_{\eta\alpha lm} = -\sum_{p'l'm'}[M^{-1}]^{(pp')}_{\alpha lm\,\alpha' l' m'}\mathcal{W}^{(p')}_{I\eta\alpha' l' m'} \quad \text{(S11)}$$

relates the multipole amplitudes of the incident field to those of the field scattered by the whole object. In order to define the T-matrix for the whole aggregate it is necessary to express the scattered field in terms of multipole fields with the same origin. Thanks to the addition theorem, the scattered field can be written as:

$$\mathbf{E}_{S\eta} = \left[\sum_{plm}\sum_{\alpha'}\sum_{p'l'm'}\mathbf{H}^{(p)}_{lm}(r_\alpha, k)J^{(pp')}_{0lm\,\alpha' l' m'}A^{(p')}_{\eta\alpha' l' m'}\right] = \sum_{plm}\mathbf{H}^{(p)}_{lm}(\mathbf{r}, k)\mathcal{A}^{(p)}_{\eta\alpha lm} \quad \text{(S12)}$$

which is valid in the region external to the smallest sphere including the whole aggregate. The previous equation shows that the field scattered by the whole cluster can be expanded as a series of vector multipole fields with a single origin (monocentered expansion) provided that the amplitudes are:

$$\mathcal{A}^{(p)}_{\eta\alpha lm} = \sum_{\alpha' p' l' m'}J^{(pp')}_{0lm\,\alpha' l' m'}A^{(p')}_{\eta\alpha' l' m'} \quad \text{(S13)}$$

Substituting for the amplitudes A their expression (S11), we can define the T-matrix of the aggregate as:

$$S^{(pp')}_{lml'm'} = -\sum_{\alpha\alpha' qq' ll' mm'}J^{(pq)}_{0lm\alpha LM}\,[M^{-1}]^{(qq')}_{\alpha LM\,\alpha' L' M'}J^{(q'p')}_{\alpha L' M' 0 l' m'} \quad \text{(S14)}$$

These are the key quantities needed to define the normalized scattering amplitude matrix of the aggregate [4] that encompasses all the observable features of the scattering process, as it is related to the flux of the electromagnetic energy that the particle scatters within the unit solid angle around the direction of observation $\hat{\mathbf{k}}_s$.

In order to calculate the T-matrix of a cluster we have to solve the system (S9) which has, in principle, infinite order. The system must be truncated to some finite order by including into the multipole expansions terms up to order l_M, chosen to ensure the convergency of the calculations. For a cluster of N_c spheres this implies solving a system of order d_M=2 N_c l_M (l_M +2), which may grow rather large. In our fiber calculations we used l_M=4 that for the largest cluster, with 360 subunits, yields $d_M = 17280$. Indeed, the inversion of the matrix M is responsible for most of the CPU time required for the calculation, which scales as d_M^3. We also note that, for our fiber sample, N_c=$N_1 n_l$ where N_1~36 is the average number of subunits composing one layer, and n_l is the number of layers. As a consequence, the computational demand increases with the cube of the number of the spheres, N_c^3, and hence with the cube of the number of layers, n_l^3. For the largest cluster the computing request was about 12 GB of RAM per single simulation, taking about 8 hours on an INTEL2650v3 CPU-based computing cluster.

Scattering mean free path and transport length. T-matrix calculations can be used to investigate the light transport properties in nanofiber samples, for which the single-scattering approximation is no more valid. Indeed this analytical approach, as clarified by Mishchenko [13], allows the radiative transfer equation to be derived from first principles. The need to consider the multiple-scattering processes has been stressed by Ishimaru and Kuga [14] who found that, as density increases, measurements might significantly depart from those expected for low-density dispersions. The multiple scattering processes within a dispersion can be included through the Foldy–Twersky equation for the propagation of the coherent field [15, 16], excluding processes that imply cyclic paths (Twersky approximation). When the primary incident field is a plane wave, the number density is a constant, and the particles are in each other's wave zone an interesting solution of the Foldy–Twersky equation can be found [17, 19] in terms of the complex forward-scattering amplitude of the aggregate. As regards the propagation of the coherent field, the fiber samples behaves as a homogeneous medium with a complex refractive index. Note that, even if the spherical particles that constitute the aggregate have a real refractive index and are therefore non-absorptive, the refractive index defined through this theoretical approach is complex, so that the dispersion as a whole behaves like an absorbing medium [17].
Thus, we can calculate the albedo at λ=510 nm for systems with increasing thickness. Results from T-matrix calculations for each system are shown as open circles in Figure S10. Thicker samples yield an albedo increasing towards the saturation value of A=1, for which the light is totally back-scattered. Following the approach given by Mishchenko *et al*. [7], a simple model can be used to follow the albedo dependence on sample thickness, that considers an exponential extinction according to the Lambert-Beer relation. In fact, we can consider a sigmoid dependence $A(z)=A_0\exp(z/l^*)/[1-A_0+A_0\exp(z/l^*)]$, where the intercept at zero thickness, A_0=0.668, is fixed so that the value for the single layer (with thickness = 700 nm) matches the T-matrix calculations (A_1=0.71) and $l^*= l_s/(1-g)$=3.4 μm (shown as a dashed line in Fig. S10) is the transport length obtained from the similarity relation [19] presented in the main text. The model is shown as a blue line in Fig. S10, and it is in good agreement with the T-matrix calculations (open circles). One can conclude that light is highly extinct within few microns of propagation in the sample and for a thickness of 25 μm the albedo is very close to unity.

Experimental albedo values. The experimental albedo is obtained as the ratio of the normalized backscattered intensity (integrated over the backscattering angle) I_{exp}, and extinction, that is expressed in terms of transmission, $E_{exp}=1-Tr$=0.996. The integrated backscattered intensity was measured through integrated sphere measurements at λ=510 nm (I_{exp}=0.773). This yields a measured albedo, A_{exp}=0.8, for samples with thickness ≅ 25 μm, that also approaches unity though being smaller than the corresponding calculated value (blue line in Fig. S10). This discrepancy can be justified by the experimental non-woven size which is much larger than the *x-y* size of modelled structure (10×10 μm^2), and by the fact that during measurements a part (~3%) of light is lost in the backward direction, since the used integrating sphere does not collect photons in a solid angle of 1.1×10^{-3} sr around the direction of the incident beam (see Methods).

Supplementary Tables

Pharmaceutical active ingredient	Commercial name	Therapeutic class	Temperature (T_c, °C)	Maximum time of exposure at T_c	Ref.
Bevacizumab	Avastin®	Anticancer antibody	30°C	9 hours	[20]
Erythropoietin alpha	Eprex®	Hormone	22-25°C	1-6 h	
Tetracosactide	Synacthen®	Diagnostic agent	< 25°C	3-4 h	
Azacitidine	Vidaza®	Anticancer	RT	45 min§	[21]
Penicillin G	Bicillin® L-A	Antibiotic	25°C	7 days	[22]
			40°C	1 day	
Insulin	Insulin regular-isophane Humulina®	Hormone	30°C	1 day	[20]
			35°C	12 hours	
Herpes zoster vaccine	Zostavax®	Vaccine	RT	30 min§	[23]
Measles, mumps, rubella and varicella (MMRV) virus vaccine	ProQuad®	Vaccine	20-25°C	30 min§	[23,24]
Rotavirus vaccine	RotaTeq®	Oral vaccine	26-30 °C	12 hours	[25]
			> 30 °C	To discard immediately	

§ Referred to the reconstituted suspension

Table S1 | Storage data reported in the literature for some pharmaceutical products.

**Bacteria**	**Disease**	**Growth temperature (T_c, °C)**	**Maximum time of exposure at T_c**	**Ref.**
Campylobacter jejuni	Campylobacteriosis	30-34°C	48 hours	[26]
		> 34°C	12 hours	
Listeria monocytogenes	Listeriosis	22-30°C	3 hours	
		T > 30°C	1 hour	
Staphylococcus aureus	Bacteremia, infective endocarditis, skin and soft-tissue infections	T > 21 °C	3 hours	
Vibrio cholerae, Vibrio parahaemolyticus	Vibriosis, gastroenteritis, septicemia	22-27°C	2 hours	
		T > 27ºC	1 hour*	
Clostridium botulinum	Botulism, the toxin causes flaccid paralysis and is potentially lethal	35°C	2 hours**	[27]

*Applies to cooked, ready-to-eat foods only

** Estimated by the empirical formula, Eq. (2) in Ref. 27.

Table S2 | Food born bacterial pathogen species, and some reported activation time-temperature profile.

Supplementary References

[1] Hammacher, J. et al. Stress engineering and mechanical properties of SU-8-layers for mechanical applications. *Microsyst. Technol.* **14**, 1515-1523 (2008).

[2] Wattanachai, P. & Antonio C. Comparison of conventional and variable frequency microwave curing of SU8 photoresist: effects on the dielectric, thermal, and morphological properties. *Eng. J.* **20**, 169-186 (2016).

[3] Waterman, P. C. Symmetry, unitarity, and geometry in electromagnetic scattering. *Phys. Rev. D* **3**, 825-839 (1971).

[4] Borghese, F., Denti, P. & Saija, R. *Scattering from model nonspherical particles* (Springer-Verlag, Berlin Heidelberg, 2007).

[5] Borghese, F., Denti, P., Saija, R., Iatì, M. A. & Sindoni, O. I. Optical properties of a dispersion of anisotropic particles with non-randomly distributed orientations. The case of atmospheric ice crystals. *J. Quant. Spectrosc. Radiat. Transfer* **70**, 237-251 (2001).

[6] Iatì, M. A. et al. Porous interstellar grains. *Mon. Not. R. Astron. Soc.* **322**, 749-756 (2001).

[7] Mishchenko, M. I., Travis, L. D. & Lacis, A. A. *Scattering, absorption, and emission of light by small particles* (Cambridge University Press, Cambridge, 2002).

[8] Saija, R. et al. Beyond Mie theory: The transition matrix approach in interstellar dust modeling. *Astrophys. J.* **559**, 993-1004 (2001).

[9] Borghese, F., Denti, P., Saija, R., Toscano, G. & Sindoni, O. I. Multiple electromagnetic scattering from a cluster of spheres. I. Theory. *Aerosol Sci. Technol.* **3**, 227-235 (1984).

[10] Borghese, F., Denti, P., Saija, R., Toscano, G. & Sindoni, O. I. Macroscopic optical constants of a cloud of randomly oriented nonspherical scatterers. *Nuovo Cimento B* **81**, 29-50 (1984).

[11] Moffa, M. et al. Biomineral amorphous lasers through light-scattering surfaces assembled by electrospun fiber templates. *Laser Photon. Rev.* **12**, 1700224 (2018).

[12] Yang, Z. et al. Electrospun conjugated polymer/fullerene hybrid fibers: photoactive blends, conductivity through tunneling-AFM, light scattering, and perspective for their use in bulk-heterojunction organic solar cells. *J. Phys. Chem. C* **122**, 3058-3067 (2018).

[13] Mishchenko, M.I. Vector radiative transfer equation for arbitrarily shaped and arbitrarily oriented particles: a microphysical derivation from statistical electromagnetics. *Appl. Opt*. **41**, 7114–7134 (2002).

[14] Ishimaru A. & Kuga, Y. Attenuation constant of a coherent field in a dense distribution of particles. *J. Opt. Soc. Am.* **72**, 1317–1320 (1982).

[15] Twersky, V. On propagation in random media of discrete scatterers. *Proc. Symp. Appl. Math*. **16**, 84–116 (1964).

[16] Ishimaru, A. *Wave propagation and scattering in random media* (Academic Press, New York, 1978).

[17] Newton, R.G. *Scattering theory of waves and particles* (McGraw-Hill, New York, 1966).

[18] Giusto, A. et al. Optical properties of high-density dispersions of particles: application to intralipid solutions. *App. Opt.* **42**, 4375-4380 (2003).

[19] Savo, R. et al. Observation of mean path length invariance in light-scattering media. *Science* **358**, 765-768 (2017).

[20] Parraga, L. P. et al. Thermolabile drugs. Operating procedure in the event of cold chain failure. *Farm. Hosp.* **35**, 190.e1-190.e28 (2011).

[21] Legeron, R., Xuereb, F., Djabarouti, S., Saux, M.-C. & Breilh, D. Chemical stability of azacitidine suspensions for injection after cold-chain reconstitution of powder and storage. *Am. J. Health-Syst. Pharm.* **70**, 2137-3142 (2013).

[22] Cohen, V., Jellinek, S. P., Teperikidis, L., Berkovits, E. & Goldman, W. M. Room-temperature storage of medications labeled for refrigeration. *Am. J. Health-Syst. Pharm.* **64**, 1711-1715 (2007).

[23] Plotkin, S. A., Orenstein, W. A. & Offit, P. A. *Vaccines, 6th Edition* (Elsevier Saunders, Philadelphia, 2012).

[24] McLean, H. Q., Fiebelkorn, A. P., Temte, J. L. & Wallace, G. S. Prevention of measles, rubella, congenital rubella syndrome, and mumps, 2013: summary recommendations of the Advisory Committee on Immunization Practices (ACIP). *Morb. Mortal. Wkly. Rep.* **62**, 1-34 (2013).

[25] World Health Organization. Detailed review paper on rotavirus vaccines. https://www.who.int/immunization/sage/3_Detailed_Review_Paper_on_Rota_Vaccines_17_3_2009.pdf?ua=1 (2009).

[26] Food and Drug Administration. Fish and fishery products hazards and controls guidance. https://www.fda.gov/media/80637/download (2011).

[27] Skinner, G. E. & Larkin, J. W. Conservative prediction of time to Clostridium botulinum toxin formation for use with time-temperature indicators to ensure the safety of foods. *J. Food Prot.* **61**, 1154-1160 (1998).
