# Supplementary material for: Intelligent non-colorimetric indicators for the perishable supply chain by non-wovens with photo-programmed thermal response
Source: Nat Commun. 2020 Nov 25;11:5991. doi: 10.1038/s41467-020-19676-y (PMC7688929; doi:10.1038/s41467-020-19676-y)
Supplement: Supplementary file 2 — Description of Additional Supplementary Files [file 41467_2020_19676_MOESM2_ESM.pdf]

Description of Additional Supplementary Files

File Name: Supplementary Movie 1
Description: Device displaying a warning sign upon heating at a temperature of 55°C.
